# Effect of Body Surface Area on Severe Osteoporotic Fractures: A Study of Osteoporosis in Changsha China

**DOI:** 10.3389/fendo.2022.927344

**Published:** 2022-07-22

**Authors:** Xi-Yu Wu, Hong-Li Li, Yi Shen, Li-Hua Tan, Ling-Qing Yuan, Ru-Chun Dai, Hong Zhang, Yi-Qun Peng, Zhong-Jian Xie, Zhi-Feng Sheng

**Affiliations:** ^1^ Hunan Provincial Key Laboratory of Metabolic Bone Diseases, National Clinical Research Center for Metabolic Diseases, Department of Metabolism and Endocrinology, The Second Xiangya Hospital of Central South University, Changsha, China; ^2^ Department of Endocrinology, The First Hospital of Lanzhou University, Lanzhou, China; ^3^ Department of Orthopaedics, The Second Xiangya Hospital, Central South University, Changsha, China; ^4^ Department of Radiology, The Second Xiangya Hospital, Central South University, Changsha, China

**Keywords:** osteoporosis, osteoporotic fracture, body surface area, bone mineral density, fracture risk

## Abstract

Clinical vertebral fractures and femoral neck fractures are severe osteoporotic fractures that increase morbidity and mortality. Anthropometric variables are associated with an increased risk of osteoporotic fractures, but it is not clear whether body surface area (BSA) has an effect on clinically severe osteoporotic fractures. The study included total of 3,694 cases of clinical vertebral fractures and femoral neck fractures (2,670 females and 1,024 males) and 3,694 controls without fractures who were matched with the cases by sex and age. There was a significant positive correlation between BSA and bone mineral density (BMD) in female and male fracture patients (females: *r* = 0.430–0.471, *P* < 0.001; males: *r* = 0.338–0.414, *P* < 0.001). There was a significant systematic increase in BMD in both genders at various skeletal sites, grouped by BSA quartile. The osteoporosis rates of the lumbar spine (97.9%), femoral neck (92.4%) and total hip (87.1%) in the female Q1 group were significantly higher than those in the Q4 group (*P* < 0.001), which were 80.0%, 57.9% and 36.9%, respectively, in the Q4 group; the osteoporosis rates of the lumbar spine, femoral neck, and total hip were 53.9%, 59.4%, and 36.3% in the male Q1 group, and 15.2%, 21.9%, and 7.03% in the Q4 group, which were significantly lower than those in the Q1 group (*P* < 0.001). In age-adjusted Cox regression models, the risk of fracture in the remaining three groups (Q2, Q3, and Q4) for weight, BMI, and BSA for both genders, compared with the highest quartile (Q1 by descending quartile stratification) were significantly higher. In models adjusted for age and BMD, only men in the BSA Q3 (HR = 1.55, 95% CI = 1.09–2.19) and BSA Q4 groups (HR = 1.41, 95% CI = 1.05–1.87) had significantly higher fracture risks. In models adjusted for age, height, weight, BMI, and BSA, low BMD was the greatest fracture risks for both sexes. Our results showed that BSA was closely related to BMD, prevalence of osteoporosis, and fracture risk, and that a decline in BSA may be a new potential risk factor for osteoporotic fractures in Chinese men.

## Background

Osteoporosis is a systemic bone disease that is characterized by a decrease in bone mass, a deterioration of the microstructure of bone tissue, and a decrease in bone strength, leading to an increase in bone fragility and susceptibility to fractures (1). Clinical vertebral and femoral neck fractures are severe osteoporotic fractures that result in increased disability, morbidity, and mortality (2–11) higher healthcare costs (11–15), and affect health-related quality of life (16–21). Although studies have shown a very low incidence of osteoporotic fractures in the Chinese mainland population (22), the incidence of osteoporotic fractures is increasing rapidly with the urbanization and aging of the Chinese population (23). It is estimated that by 2050, half of the world’s osteoporotic fractures will occur in Asia, primarily in China (24). As a result, osteoporotic fractures will become an even more serious public health problem in the Chinese mainland.

It is well known that low bone mineral density (BMD) is an important risk factor for osteoporotic fractures (24, 25), but there are many other risk factors for osteoporotic fractures besides BMD (11, 26, 27), such as age, sex, height, weight, body mass index (BMI), past fragility fractures, long-term glucocorticoid, a history of falls, parental hip fractures, long-term smoking, long-term drinking, rheumatoid arthritis, dementia, and various types of secondary osteoporosis. Therefore, most fragility fractures occur in non-osteoporotic individuals (28, 29). Studies have shown that the relationship between anthropometric indicators (height, weight, and BMI) and fracture risk varies by skeletal site, including the risk of hip fractures, clinical vertebral fractures, and wrist fracture in women, which decreases significantly with increasing BMI (30). Moreover, the risk of ankle fractures in women increases with weight gain, the risk of upper arm/shoulder and collarbone fractures decreases with height, and the risk of pelvic and rib fractures have a negative association with being underweight, and a positive association with being obese (30). A higher BMI leads to a significant increase in the risk of ankle, calf, and humerus fractures, but there is a significant decrease in hip and wrist fractures among obese women (31). A US study found that 58% of men with fractures were obese, that 62% of hip fractures and 68% of non-vertebral fractures occurred in overweight and obese men, and that a higher BMI in men was associated with an increased risk of fractures (32). Body surface area (BSA) is an anthropometric parameter that reflects body size, and our previous studies have found that age-related BSA is positively associated with BMD and the prevalence of osteoporosis at different skeletal sites in the reference population (33). However, whether BSA is associated with osteoporotic fractures is not clear. The purpose of this study was to investigate the effect of BSA, which reflects body size, on clinical vertebral fractures and femoral neck fractures, in an attempt to discover new potential risk factors for the prevention of clinically severe osteoporotic fractures. Therefore, we decided to study the relationships of BSA and BMD with the prevalence of osteoporosis in patients with clinically severe osteoporotic fractures, and the effect of BSA on severe osteoporotic fractures.

## Materials and Methods

### Participants

The study was conducted from March 2011 to October 2021 at the Second Xiangya Hospital of Central South University, Changsha, China. Patients diagnosed with osteoporotic fractures by imaging were considered potential subjects for the case group. The inclusion criteria for severe osteoporotic fractures were patients who came to the hospital with symptoms of vertebral fractures or femoral neck fractures, patients who reported low-injury fractures that occurred from falling from a standing height or less, or occurred without falling. A vertebral body fracture was confirmed by a radiologist based on a lateral vertebral radiograph and a femoral neck fracture was confirmed a by radiologist based on a proximal femoral radiograph, using semi-quantitative methods (34). Patients were excluded from the study if their fractures were caused by trauma (such as a car accident or a fall from a chair or higher) or they had local pathological fractures caused by cancer, bilateral hip fractures, non-vertebral fractures, or non-femoral neck fractures. A total of 3,694 patients with severe osteoporotic fractures met the inclusion criteria, including 2,670 women, who were 40–94 years-old and had a mean (± SD) age of 67.5 ± 8.61 years, and 1,024 men, who were 40–100 years-old and had a mean age of 65.8 ± 12.4 years. These patients had 3,181 vertebral fractures (2,296 females and 885 males) and 513 femoral neck fractures (374 females and 139 males).

The data of 3,694 patients assigned to the control group were obtained from a reference population of a BMD database, which was established by us before the study (35, 36). A 1:1 ratio between the control group and the case group was used, according to sex and age. The inclusion criterion for the control group was having no history of a low- or a high-injury fracture, and the exclusion criteria were osteosclerosis, skeletal fluorosis, or abnormally increased BMD. This study was approved by the Ethics Committee of Second Xiangya Hospital affiliated with the Central South University. All the participants were of Han ethnicity.

### BMD Measurement

The lumbar spine (L1–L4), femoral neck, and total hip BMDs were measured by fan-beam dual-energy X-ray (DXA) absorptiometry (Hologic Delphi A; Hologic, Bedford, MA, USA). If the lumbar vertebrae of patients with vertebral body fractures were filled with postoperative artificial bone cement or contained installed metal brackets, these lumbar vertebrae were excluded from the analysis. The right hip was measured if the patient had a left femoral neck fracture or had a hip replacement. If patients had bilateral femoral neck or hip fractures, the hip measurements were discarded and these patients were excluded from the study. BMD was measured twice in 33 subjects. The root-mean-square coefficients of precision (root-mean-square CV; RMSCV) were 0.86%, 1.17%, and 0.88% for the lumbar spine, femoral neck, and total hip, respectively. The long-term (> 17 years) CV of routine quality control phantom measured daily by DXA bone densitometer was < 0.45%. Using our own BMD reference database for women and men (35, 36), we calculated the sex-specific BMD T-score of the lumbar spine, femoral neck, and total hip. According to the World Health Organization (WHO) definition (37), participants with a T-score > −1.0 had normal BMD; those with a −2.5 < T-score ≤ −1.0, whereas those with a T-score ≤ −2.5, when compared with the same sex peak BMD, were classified as having osteopenia and osteoporosis, respectively.

### BSA Estimation and BMI Classification

BSA was estimated based on the average height and weight of Chinese adults (38); its estimation formula for males was BSA = 79.8106 × H^0.7271^ × W^0.3980^; and its estimation formula for females was BSA = 84.4673 × H^0.6997^ × W^0.4176^; where BSA was expressed in cm^2^, height (H) in cm, and body weight (W) in kg. According to the BMI classification criteria for overweight and obesity in Chinese adults (39), a BMI < 18.5 kg/m^2^ was considered a low body weight, a BMI = 18.5–23.9 kg/m^2^ was considered a normal body weight, a BMI = 24.0–27.9 kg/m^2^ was considered overweight, and a BMI ≥ 28.0 kg/m^2^ was considered obese.

### Statistical Analysis

All analyses were performed using SPSS V23.0 for Windows (SPSS Inc., Chicago, IL, USA). Kolmogorov-Smirnov test (K-S test) was used to explore normal distribution of the data. The K-S test results showed that the age, height and weight of the subjects of both genders and the age at menopause (AM) and years since menopause (YSM) of women did not meet the normal distribution criteria (Z = 1.495–2.471, *P* = 0.023 to < 0.001), the rest of the indicators (BMI, BSA and BMD) basically met the normal distribution standard (Z = 0.466–1.306, *P* = 0.982–0.066). The indicators that did not meet the standard of normal distribution were expressed by median and range. If there was a significant difference between groups, test for two independent samples was used. Indicators meeting the normal distribution criteria were expressed as mean and standard deviation (SD) and one-way analysis of variance (ANOVA). The relationship between BSA and BMD at various skeletal sites was analyzed using Pearson’s correlation. The patients in the case group were divided into quartiles according to their BSA, and the differences in mean BMD, prevalence of osteoporosis, and fracture risks were compared among these four subgroups. The relationships of different variables with the risk of osteoporotic fracture were analyzed by multivariate Cox regression models, which produced multivariate hazard ratios (HRs) for fractures and their 95% confidence intervals (CIs). The multivariate analysis included adjustments for age, height, weight, BMI, and BSA or BMD. The differences in the prevalence of osteoporosis and osteopenia between genders and across different groups of fracture patients were compared using the chi-square test. A *P* < 0.05 was considered statistically significant.

## Results

### Characteristics of the Participants

The rates of obesity, overweight, and normal BMI in fracture patients were, respectively, 5.24%, 28.6%, and 56.0% for females, and 4.88%, 23.0%, and 60.7% for males. **Table 1** showed that the median age of each sex in the case group was exactly the same as that of the control group, as the sex and age of the case group were exactly the same as the control group. In both sexes, the median height, weight, BMI, BSA, and BMD at each bone site in the case group were significantly lower than the medians in the control group. The median age at menopause (AM) of females in the case group was significantly younger than the median of females in the control group, and the median years since menopause (YSM) was significantly older in the case group than that in the control group. The median age and YSM of females in the single vertebral fracture (SVF) subgroup were significantly lower than those in multiple vertebral (2 or more) fracture (MVF) and multiple sites fracture (MSF) subgroups, while their FN-BMD and Hip-BMD were significantly higher than those in the MVF and MSF subgroups (**Table 1**). The median height, weight, and BSA of the females in the SVF group were significantly higher than the medians of the females in the MVF group. The median age, SYM, height, weight, BSA, and LS-BMD of the MVF group were significantly lower than the medians of the MSF group. Among the male cases, the median age of the SVF subgroup was significantly lower than the medians of the MVF and MSF subgroups, and their FN-BMD and Hip-BMD were significantly higher than those of the MVF and MSF subgroups (**Table 1**). The median weight, BMI, and BSA of the males in the SVF subgroup were significantly higher than the medians in the MVF subgroup. The median age, height, BSA, and LS-BMD in the MVF subgroup were significantly lower than the medians in the MSF subgroup.

**Table 1:** Comparison of basic characteristics among cases of fractures and controls.

Parameter		Control	Case		Fracture subgroup				
					SVF		MVF		MSF
**Female**									
*n* (%)		2670	2670		855 (32.0)	1201 (45.0)		614 (23.0)	
Age (years)^a^		68.0 (40–94)	68.0 (40–94)		67.0 (40–93)^cd^	68.0 (40–94)^f^		70.0 (40–93)^c^	
AM (years)^a^		50.0 (40–64)	49.0 (40–60)^b^		48.0 (40–58)^c^	49.0 (40–60)		49.0 (40–59)	
YSM (years)^a^		18.0 (1–54)	19.0 (1–47)^b^		18.0 (1–43)^cd^	19.0 (1–44)^f^		21.0 (1–47)^cd^	
Height (cm)^a^		152.0 (134–170)	150.0 (112–173)^b^		151.5 (130–172)^ce^	149.0 (121–173)^cf^		152.0 (112–173)^c^	
Weight (kg)^a^		55.0 (30–94)	51.0 (26–93)^b^		52.0 (26–82.5)^ce^	50.0 (28–93)^cf^		52.0 (31–75)^c^	
BMI (kg/m^2^)		23.8 ± 3.46	22.7 ± 3.33^b^		22.7 ± 3.38	22.6 ± 3.42		22.7 ± 3.04	
BSA (m^2^)		1.51 ± 0.12	1.46 ± 0.13^b^		1.48 ± 0.12^ce^	1.44 ± 0.13^cf^		1.47 ± 0.12^c^	
LS-BMD (g/cm^2^)		0.760 ± 0.136	0.620 ± 0.110^b^		0.635 ± 0.099^cd^	0.592 ± 0.109^cf^		0.654 ± 0.115^c^	
FN-BMD (g/cm^2^)		0.612 ± 0.109	0.507 ± 0.093^b^		0.529 ± 0.086^cd^	0.496 ± 0.098^c^		0.498 ± 0.089^c^	
Hip-BMD (g/cm^2^)		0.688 ± 0.127	0.585 ± 0.114^b^		0.615 ± 0.105^cd^	0.570 ± 0.119^c^		0.573 ± 0.109^c^	
**Male**									
*n* (%)	1024		1024	381 (37.2)		436 (42.6)		207 (20.2)	
Age (years)^a^	66.0 (40–100)		66.0 (40–100)	62.0 (40–100)^cd^		66.0 (40–95)^f^		72.0 (40–96)^c^	
Height (cm)^a^	165.0 (142–188)		163.0 (132–180)^b^	163.0 (132–180)^f^		162.0 (142–179)^cf^		165.0 (142–178)^c^	
Weight (kg)^a^	67.0 (34–107)		59.0 (30–100)^b^	60.0 (30–100)^e^		56.0 (31–91)^c^		60.0 (36–90)	
BMI (kg/m^2^)	24.6 ± 3.19		22.4 ± 3.31^b^	22.6 ± 3.18^e^		21.9 ± 3.26		22.3 ± 3.59	
BSA (m^2^)	1.74 ± 0.13		1.64 ± 0.15^b^	1.65 ± 0.14^e^		1.61 ± 0.15^cf^		1.66 ± 0.15^c^	
LS-BMD (g/cm^2^)	0.971 ± 0.148		0.720 ± 0.120^b^	0.731 ± 0.088^d^		0.687 ± 0.117^cf^		0.770 ± 0.153^c^	
FN-BMD (g/cm^2^)	0.741 ± 0.123		0.582 ± 0.102^b^	0.599 ± 0.087^cd^		0.577 ± 0.102		0.561 ± 0.122^c^	
Hip-BMD (g/cm^2^)	0.875 ± 0.133		0.691 ± 0.124^b^	0.714 ± 0.111^cd^		0.677 ± 0.125^c^		0.678 ± 0.138	

a Values are median (range). Other values are mean ± SD.

AM, age at menopause; YSM, years since menopause; BMI, body mass index; BSA, body surface area; LS, lumbar spine; BMD, bone mineral density; FN, femoral neck; Hip, total hip; SVF, single vertebral fracture; MVF, multiple vertebral (2 or more) fracture; MSF, multiple sites fracture (vertebral accompany other sites and femoral neck fractures).

b P = 0.014 to < 0.001 compared with control.

c P = 0.045 to < 0.001 compared with case.

d P = 0.008 to < 0.001 compared with MVF and MSF.

e P = 0.002 to < 0.001 compared with MVF.

f P = 0.005 to < 0.001 compared with MSF.

Among the cases (**Table 2**), the prevalence of osteoporosis in the lumbar spine, femoral neck, and total hip were, respectively, 89.6%, 76.6%, and 61.9% in female cases and 31.2%, 39.6%, and 19.7% in male cases. The rate of osteoporosis was significantly higher in female cases than male cases, with the majority of female cases suffering from osteoporosis. The rate of low bone mass in the lumbar spine, femoral neck and total hip were, respectively, 9.33%, 21.7%, and 34.1% in female cases and 58.5%, 56.9%, and 64.5% in male cases, and the rates of low bone mass and normal BMD were also significantly higher in male cases than female cases.

**Table 2:** Number and rates of osteoporosis, osteopenia and normal BMD in fractures using gender specific *T*-scores.

Skeletal site	Female (*n* = 2670)			Male (*n* = 1024)		
	Osteoporosis*n* (%)	Osteopenia*n* (%)	NBMD*n* (%)	Osteoporosis*n* (%)^c^	Osteopenia*n* (%)^c^	NBMD*n* (%)^c^
Lumbar spine	2393 (89.6)^a^	249 (9.33)^a^	28 (1.05)^b^	319 (31.2)^a^	599 (58.5)^e^	106 (10.4)^a^
Femoral neck	2044 (76.6)^b^	579 (21.7)^b^	47 (1.76)^b^	406 (39.6)^b^	583 (56.9)^be^	35 (3.42)^b^
Total hip	1652 (61.9)	911 (34.1)	107 (4.01)	202 (19.7)	660 (64.5)^e^	162 (15.8)

NBMD, normal bone mineral density.

a P = 0.006 to < 0.001 compared with femoral neck and total hip on same parameter.

b P = 0.001 to < 0.001 compared with total hip on same parameter.

c P = 0.003 to < 0.001 compared with female on same parameter.

e P < 0.001 compared with osteoporosis on same parameter.

### Association of BSA With the BMD and Prevalence of Osteoporosis

**Figure 1:**
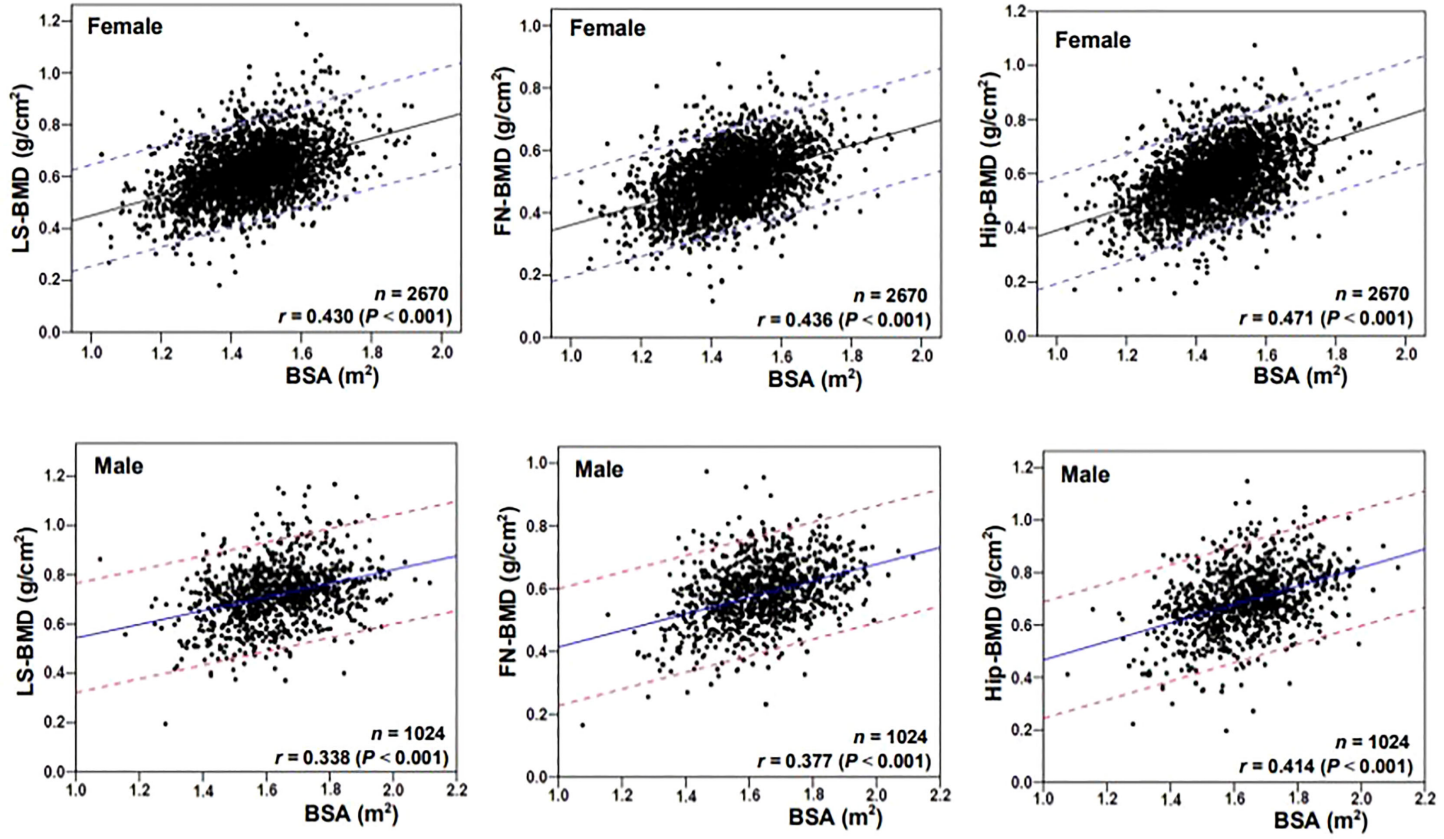
Correlation scatter diagrams of the body surface area (BSA) with BMD at various skeletal sites. LS, lumbar spine; BMD, bone mineral density; FN, femoral neck; Hip, total hip.

**Figure 2:**
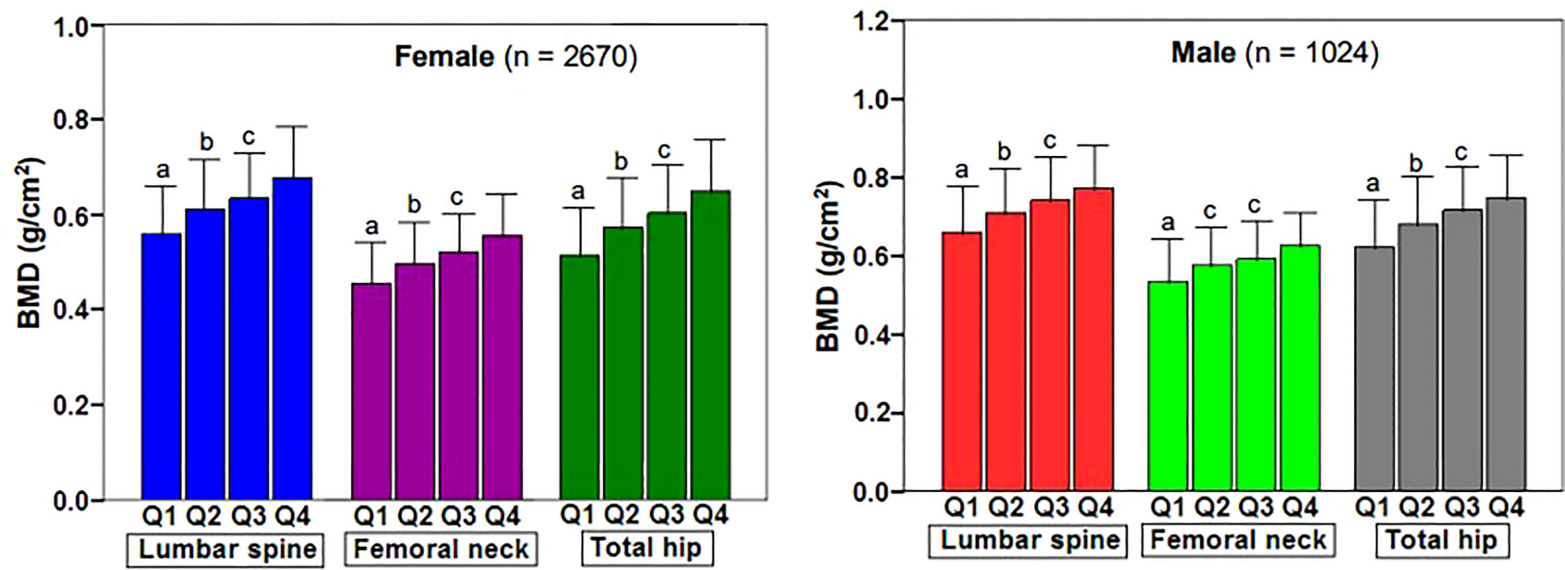
Changes of BMD at various skeletal sites in body surface area of fracture patients stratified by quartile. BMD, bone mineral density; Q1, first quartile; Q2, second quartile; Q3, third quartile; Q4, fourth quartile; LS, lumbar spine (L1–L4); FN, femoral neck; TH, total hip. ^a^ *P* < 0.001 compared with Q2, Q3 and Q4. ^b^ *P* = 0.001 to < 0.001 compared with Q3 and Q4. ^c^ *P* = 0.003 to < 0.001 compared with Q4.

**Figure 3:**
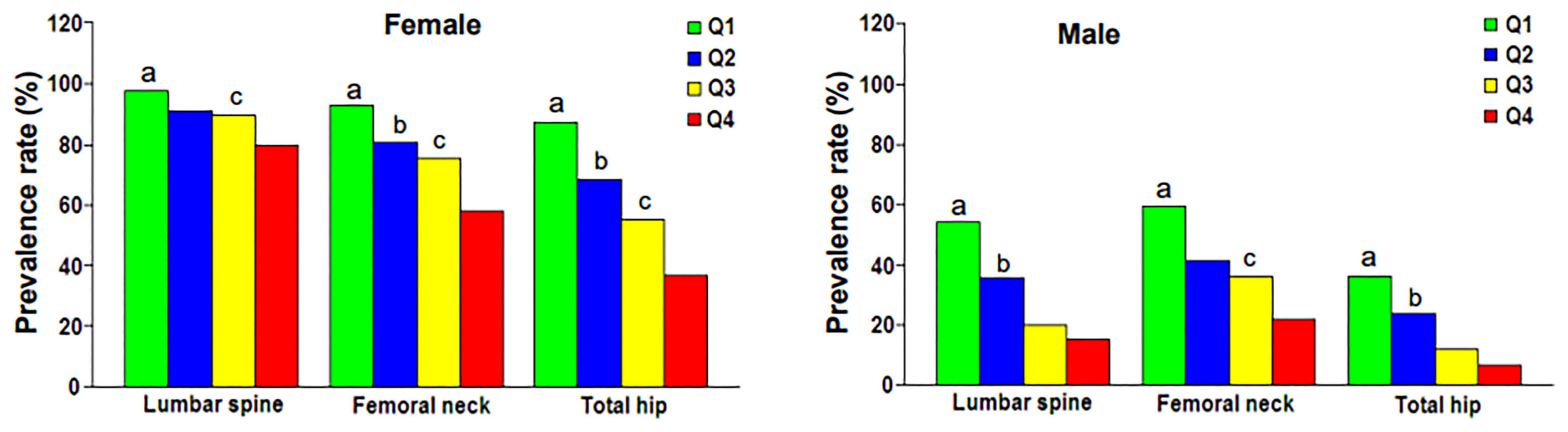
Prevalence rate of osteoporosis at various skeletal regions according to body surface area quartile. Q1, first quartile; Q2, second quartile; Q3, third quartile; Q4, fourth quartile. ^a^ *P* = 0.003 to < 0.001 compared with Q2, Q3 and Q4. ^b^ *P* = 0.028 to < 0.001 compared with Q3 and Q4. ^c^ *P* = 0.001 to < 0.001 compared with Q4.

**Figure 1** showed the correlation between BSA and BMD in the case group by sex and skeletal site. BSA had a significant positive correlation with BMD in females and males, but the correlations between BSA and BMD (*r* = 0.430–0.471, *P* < 0.001) were higher for females than they were for males (*r* = 0.338–0.414, *P* < 0.001). The correlation of BSA with Hip-BMD was higher than the correlation of BSA with LS-BMD and FN-BMD. **Figure 2** showed the BSA of the case group stratified into quartiles, and compared the mean BMD of each BSA quartile for three skeletal sites. The analyses of the BMD of males and females found BMD exhibited a significant positive trend across BSA quartiles in both sexes at each site; that was, Q1<Q2<Q3<Q4. **Figure 3** showed the BSA of the case group stratified into quartiles, and compared the prevalence of osteoporosis for each BSA quartile. The analyses found that the prevalence of osteoporosis was highest when BSA was lowest (Q1) for both sexes at all three skeletal sites. The lowest BSA quartile (Q1) had the highest prevalence and highest BSA quartile (Q4) had the lowest prevalence for both sexes at all three sites. However, only the female femoral neck and total hip showed significant sequential decreases in the prevalence of osteoporosis across quantiles; that was, prevalence exhibited a trend of Q1>Q2>Q3>Q4.

### Fracture Hazard Ratios

**Table 3** showed the fracture hazard ratios (HRs) of seven variables with the anthropometric index and BMD for each quartile of each variable (Q1 = highest to Q4 = lowest) based on multivariate Cox regression. In the age-adjusted models, regardless of sex, the fracture hazard ratios (HR1) for weight, BMI, BSA, LS-BMD, FN-BMD, and Hip-BMD were significantly higher in the Q2, Q3, and Q4 groups (*P* = 0.019 to < 0.001) than the reference group (Q1); only the female Q4 group with the smallest height (height ≤ 147.9 cm) had a significantly higher HR1 (HR1 = 1.38, *P <* 0.001); HR1 also was significantly higher in the Q3 and Q4 groups of males. In the models adjusted for age and BMD, the increases in HR2 for each quantile of weight, BMI, and BSA of females were no longer statistically significant, but there was a significant increase in HR2 for the height Q4 group of females (HR2 = 1.12, *P* = 0.017). The HR2 was also significantly higher in the BSA Q3 (HR2 = 1.55, *P* = 0.015) and BSA Q4 (HR2 = 1.41, *P* = 0.020) groups of males. In the models adjusted for age, height, weight, BMI, and BSA, the HR2 in the female each quantile (Q2 to Q4) varied from 2.30 to 4.42 as BMD decreased; in the male each quantile, the same measures varied from 3.48 to 8.74 (**Table 3**). **Table 4** showed the fracture HRs based on Cox regression, according to the diagnostic criteria for osteoporosis, with normal BMD as the reference. The HRs for low bone mass (LBM) and osteoporosis varied by gender and skeletal site, with the HRs of females with lumbar spine, femoral neck, and total hip osteoporosis being, respectively, about 1.6 (4.09/2.55), 2.5 (10.9/4.29), and 1.2 times (4.03/3.33) higher than those for LBM, and the fracture HRs of females with osteoporosis was greater than that of females with LBM. The HRs of males with LBM for the lumbar spine, femoral neck, and total hip were approximately 3.2 (26.1/8.08), 1.1 (13.1/12.2), and 0.8 times (9.18/11.7) higher, respectively, than those for osteoporosis, and the HRs of the lumbar spine and femoral neck of males with LBM were somewhat greater than those with osteoporosis. However, the fracture HRs of the total hip of males with LBM were somewhat lower than males with osteoporosis.

**Table 3:** The effect of anthropometry and BMD stratification on fracture hazard ratio (HR).

Variable	Female (*n* = 5340)		Male (*n* = 2048)	
	HR1 (95% CI)^a^	HR2 (95% CI)	HR1 (95% CI)^a^	HR2 (95% CI)
Height Q1	Ref	Ref	Ref	Ref
Q2	0.91 (0.73–1.13)	0.66 (0.49–1.08)^b^	1.13 (0.80–1.60)	0.77 (0.34–1.79)^b^
Q3	1.03 (0.93–1.14)	0.95 (0.83–1.08)^b^	**1.25 (1.04–1.50)**	1.31 (0.87–1.96)^b^
Q4	**1.38 (1.28–1.49)**	**1.12 (1.02–1.23)** ^b^	**1.41 (1.21–1.64)**	1.25 (0.94–1.66)^b^
Weight Q1	Ref	Ref	Ref	Ref
Q2	**1.57 (1.28–1.94)**	1.06 (0.81–1.40)^b^	**2.19 (1.48–3.23)**	1.86 (0.81–4.28)^b^
Q3	**1.45 (1.29–1.61)**	0.97 (0.84–1.12)^b^	**1.67 (1.38–2.03)**	1.14 (0.77–1.69)^b^
Q4	**1.43 (1.32–1.55)**	1.06 (0.96–1.17)^b^	**2.34 (1.90–2.87)**	1.32 (0.99–1.75)^b^
BMI Q1	Ref	Ref	Ref	Ref
Q2	**1.58 (1.28–1.96)**	1.20 (0.92–1.58)^b^	**1.57 (1.10–2.23)**	1.36 (0.62–3.02)^b^
Q3	**1.34 (1.20–1.50)**	0.92 (0.80–1.07)^b^	**1.71 (1.41–2.07)**	1.00 (0.73–1.37)^b^
Q4	**1.28 (1.19–1.38)**	0.99 (0.90–1.09)^b^	**1.91 (1.62–2.25)**	1.21 (0.93–1.57)^b^
BSA Q1	Ref	Ref	Ref	Ref
Q2	**1.55 (1.24–1.93)**	1.19 (0.89–1.57)^b^	**1.80 (1.26–2.59)**	0.72 (0.35–1.48)^b^
Q3	**1.39 (1.25–1.55)**	1.04 (0.90–1.20)^b^	**2.02 (1.63–2.50)**	**1.55 (1.09–2.19)** ^b^
Q4	**1.49 (1.37–1.62)**	1.08 (0.97–1.20)^b^	**2.10 (1.72–2.56)**	**1.41 (1.05–1.87)** ^b^
LS-BMD Q1	Ref	Ref	Ref	Ref
Q2	**4.18 (3.12–5.61)**	**4.17 (3.11–5.60)** ^c^	**9.14 (4.19–19.9)**	**8.74 (3.98–19.2)** ^c^
Q3	**3.89 (3.17–4.79)**	**3.93 (3.17–4.87)** ^c^	**6.26 (4.02–9.76)**	**6.66 (4.08–10.9)** ^c^
Q4	**2.98 (2.55–3.49)**	**2.91 (2.48–3.41)** ^c^	**4.03 (2.77–5.86)**	**4.65 (3.12–8.67)** ^c^
FN-BMD Q1	Ref	Ref	Ref	Ref
Q2	**3.43 (2.62–4.49)**	**3.35 (2.55–4.40)** ^c^	**6.69 (3.96–11.3)**	**5.82 (3.41–9.92)** ^c^
Q3	**4.23 (3.39–5.26)**	**4.42 (3.51–5.56)** ^c^	**5.12 (3.40–7.71)**	**4.43 (2.92–6.73)** ^c^
Q4	**2.79 (2.39–3.27)**	**2.72 (2.32–3.19)** ^c^	**4.46 (2.80–7.09)**	**3.82 (2.39–6.11)** ^c^
Hip-BMD Q1	Ref	Ref	Ref	Ref
Q2	**2.50 (1.96–3.18)**	**2.52 (1.97–3.22)** ^c^	**3.87 (2.45–6.12)**	**3.68 (2.27–5.95)** ^c^
Q3	**2.36 (2.04–2.74)**	**2.32 (2.00–2.69)** ^c^	**4.07 (2.96–5.61)**	**3.71 (2.64–5.21)** ^c^
Q4	**2.37 (2.09–2.70)**	**2.30(2.02–2.61)** ^c^	**3.94 (2.70–5.77)**	**3.48 (2.35–5.14)** ^c^

BMI, body mass index; BSA, body surface area; LS, lumbar spine; BMD, bone mineral density; FN, femoral neck; Hip, total hip; Q1, first quartile; Q2, second quartile; Q3, third quartile; Q4, fourth quartile.

The height, weight, BMI, BSA and BMDs respectively by quartile descending stratification.

a Adjusted for age.

b Adjusted for age and BMD.

c Adjusted for age, height, weight, BMI and BSA.

Significant HRs are shown in bold.

**Table 4:** Influence of osteoporosis classification on fracture hazard ratio (HR).

Skeletal site	Female OP classification (*n* = 5340)			Male OP classification (*n* = 2048)		
	NBMD	LBM-HR (95% CI)	OP-HR (95% CI)	NBMD	LBM-HR (95% CI)	OP-HR (95% CI)
Lumbar spine	Ref	**2.55 (1.27–5.11)**	**4.09 (3.18–5.27)**	Ref	**26.1 (16.5–41.2)**	**8.08 (4.82–13.5)**
Femoral neck	Ref	**4.29 (2.84–6.47)**	**10.9 (6.17–19.2)**	Ref	**13.1 (8.28–20.6)**	**12.2 (4.55–32.5)**
Total hip	Ref	**3.33 (2.46–4.50)**	**4.03 (3.20–5.08)**	Ref	**9.18 (6.86–12.3)**	**11.7 (4.38–31.3)**

NBMD, normal bone mineral density; LBM, low bone mass (osteopenia); OP, osteoporosis.

Significant HRs are shown in bold.

## Discussion

This paper reported the results of a sex- and age-matched case-control study, in which patients with clinically severe osteoporotic vertebral fractures, and femoral neck fractures served as cases, and the control group was a reference population (35, 36) without any fractures. We found that anthropometric indicators (height, weight, BMI, and BSA) and BMD at various skeletal sites were associated with fracture risks that were significantly lower in both genders in the case group than in the control group, suggesting that the overall decrease in these parameters may be the direct cause of fractures. Regardless of gender, the MVF subgroup of the case group had the lowest BMD, which may be an important cause of MVFs. Due to multiple vertebral compression fractures, the height of the MVF subgroup was significantly lower in both genders. In the MSF subgroup, the proportion of patients with femoral neck fractures was greater (60.9% in women and 67.1% in men), and the majority of femoral neck fractures occurred in older adults; as a result, the age of both women and men in the MSF subgroup were significantly higher.

Our study showed that female patients with clinically severe osteoporotic fractures had very high rates of osteoporosis in the lumbar spine (89.6%) and total hip (61.9%), whereas the rates in male patients were very low (only 31.2%% and 19.7%, respectively), and about 2.87 times (89.6/31.2) and 3.14 times (61.9/19.7) higher in women than in men, respectively, yielding significant differences between the sexes. This suggested that severe osteoporotic fractures occur in only a small proportion of females and a large proportion of males who did not have osteoporosis. Our results were similar to those of previous research that found the osteoporotic rate of female fracture patients was significantly higher than that of males (28). The research literature also showed that 44% of non-vertebral fractures and 64% of hip fractures occurred in osteoporotic women, compared to roughly 21% and 39% (28) in men, respectively. In contrast, the majority of osteoporotic fractures in postmenopausal women (approximately 60–82%) were found to occur in individuals with low bone mass and normal BMD (29, 40, 41), and this was attributed to the fact that the proportion of individuals with low bone mass and normal BMD was much higher than the proportion of individuals with osteoporosis (40). Based on the Chinese adult obesity standard (BMI ≥ 28.0 kg/m^2^ was considered obese) (39), the obesity rates of the female and male fracture patients in this study were 5.2% and 4.9%, respectively, and the obesity rate was significantly lower than that of 37.5% of women and 58% of men with fractures in North America (32, 42), while the obesity rate of Chinese was only 13.9% (5.2/37.5) and 8.4% (4.9/58) of North American women and men, respectively, which suggested there was a significant racial difference.

We found BSA was strongly associated with BMD, prevalence of osteoporosis, and fracture risk in male and female cases. BSA was significantly and positively associated with lumbar spine, femoral neck, and total hip BMD in cases of both genders (**Figure 1**), and when BSA levels were stratified by ascending quartile, the mean BMD increased significantly (**Figure 2**) from group Q1 (lowest BSA levels) to group Q4 (highest BSA level), while the prevalence of osteoporosis decreased significantly (except for the female lumbar spine) (**Figure 3**). This indicated that BMD increased with increased BSA, whereas the prevalence of osteoporosis decreased with increased BSA. The relationship between BSA and BMD in patients with fractures and its effect on the prevalence of osteoporosis in this study were similar to our previous study of a female reference population (33). When BSA levels were stratified by quartiles in descending order (taking the Q1 group with the highest BSA levels as a reference), an age-adjusted model found as BSA levels decreased sequentially, the fracture risk (HR1) of women in the Q2, Q3, and Q4 groups increased non-linearly by 55%, 39%, and 49%, respectively (**Table 3**), and men had a linear increase in fracture risk of 80%, 102%, and 110%, respectively. In models adjusted for age and BMD, weight, BMI, and BSA in women, and height, weight, and BMI in men were not significantly associated with fracture risk (HR2), suggesting that these anthropometric indicators were not independent factors of BMD for fracture risk. However, in men, even after adjusting for age and BMD, the fracture risk remained significantly higher in Q3 (HR2 = 1.55) and Q4 (HR2 = 1.41) groups, which suggested that BSA may be a risk factor for clinically severe osteoporotic fractures in men, independent of age and BMD. But BSA was not an independent risk factor for fractures in women. The main reason for the gender differences in the relationship between fracture risk and BSA was that there may be a very complex relationship between HR of fractures and BSA, whether female or male. Secondly, the prevalence of osteoporosis in the female fracture group was about 3 times that of the male group, indicating that the female fracture group lost more BMD and the male fracture group had less BMD loss, and the female fracture group was affected by BMD much more than the male group. Therefore, after adjusted for BMD, the effect of BSA on fracture risk in women was decreased or disappeared, and the effect on fracture risk in men was attenuated decreased but still significant. In this study, the BSA stratification of men was BSA = 1.5895–1.6895 m^2^ in group Q3 and BSA ≤ 1.5892 m^2^ in group Q4 (the results were not shown), so we determined when BSA ≤ 1.6895 m^2^, the risk of severe osteoporotic fracture in men was significantly increased by about 41–55%.

Our study also showed when BMD was stratified by descending quartiles (highest in Q1, lowest in Q4, with Q1 as the reference), and two models that adjusted for age (HR1) or adjusted for age, height, weight, BMI, and BSA (HR2) (**Table 3**), the BMD of the lumbar spine in both sexes and the femoral neck in men decreased gradually with increasing quartiles (from Q2 to Q4), but the fracture HR did not increase with BMD, and decreased linearly, such that the HR2 for Q2, Q3, and Q4 (stratified by LS-BMD) for women was 4.17, 3.93, and 2.91, respectively, and for men it was 8.74, 6.66, and 4.65, respectively. Theoretically, fracture risk should increase with decreasing BMD, but here we found the exact opposite, which was an inexplicable bizarre phenomenon. Further research is needed. However, in the osteoporosis classification (**Table 4**), osteoporotic women with a lower BMD of the lumbar spine, femoral neck, and total hip had fracture HRs that were 1.6, 2.5, and 1.2 times higher, respectively, than those for low bone mass. In contrast to women, the HRs of men with low bone mass in the lumbar spine and femoral neck were 3.2 and 1.1 times higher, respectively, than men with osteoporosis. The reason for the higher fracture risk in men with low bone mass than men with osteoporosis (except for the hip) may be related to the fact that the proportion of patients with low bone mass fractures was higher. In summary, these findings suggested that the risk of clinically severe osteoporotic fractures was associated with sex, skeletal site, and the methods used to stratify various risk factors.

This study has some limitations. First, it was not a multi-center study, and its results may only be representative of the population in and around Changsha City. Because of China’s vast territory, differences between the north and the south and between the east and the west are large, so more extensive multi-center studies are needed. Second, this study did not have a follow-up survey, and its results could not necessarily reflect causality. The third limitation is that the measure of height in patients with vertebral fractures, especially those with multiple vertebral compression fractures, may be unduly low, thereby affecting the accuracy of the BSA and BMI calculations. The BSA was not a direct measurement, but was estimated using a correlation formula based on the subject’s height and weight (38), and there may also be a risk of introducing bias. Fourth, whether BSA has the same effect on fracture risk as BMI does is skeletal site-specific and needs to be investigated further.

## Conclusion

Our study suggested that among patients with clinically severe osteoporotic fractures, the prevalence of osteoporosis in women was approximately three times that in men, and there was a significant difference between the two genders. Obesity rates among women and men with fractures were approximately 14% and 8%, respectively, of those in North American countries. In both genders, BSA was significantly positively associated with BMD in fracture patients, and the prevalence of osteoporosis decreased with increasing BSA. In models adjusted for age and anthropometric measures (height, weight, BMI and BSA), decreased BMD or osteoporosis was the greatest risk factor for fracture risk in both genders, and increased fracture risk varied with sex and BMD at different skeletal sites. In age-adjusted models, fracture risk increased non-linearly and linearly in women and men, respectively, with decreasing BSA levels. In models adjusted for age and BMD, decreased BSA remained a risk factor for increased fracture risk in men. Therefore, it suggested that lower BSA may be a new potential fracture risk factor independent of BMD in men, and its importance should be considered when assessing clinical fracture risk.

## Data Availability Statement

The original contributions presented in the study are included in the article/supplementary material. Further inquiries can be directed to the corresponding author.

## Ethics Statement

The studies involving human participants were reviewed and approved by the Ethics Committee of the Second Xiangya Hospital of Central South University. Written informed consent for participation was not required for this study in accordance with the national legislation and the institutional requirements.

## Author Contributions

Z-FS and X-YW designed the study and wrote the manuscript. X-YW, H-LL, YS, L-HT, and L-QY conducted data collection and data analysis. R-CD, HZ, Y-QP, and Z-JX acquired data from chart review and performed the analysis. Z-FS, X-YW, H-LL, YS, L-HT, L-QY, R-CD, HZ, Y-QP, and Z-JX reviewed and revised the manuscript. All the authors contributed to the article and approved the submitted version of it.

## Funding

This work was supported in part by grants from the National Natural Science Foundation of China (81500685), the Ministry of Health of the People’s Republic of China (200446850) and the Clinical Big Data Project of Central South University ([2013]15-86), China.

## Conflict of Interest

The authors declare that the research was conducted in the absence of any commercial or financial relationships that could be construed as a potential conflict of interest.

## Publisher’s Note

All claims expressed in this article are solely those of the authors and do not necessarily represent those of their affiliated organizations, or those of the publisher, the editors and the reviewers. Any product that may be evaluated in this article, or claim that may be made by its manufacturer, is not guaranteed or endorsed by the publisher.
